# Comparing Pre-trained and Feature-Based Models for Prediction of Alzheimer's Disease Based on Speech

**DOI:** 10.3389/fnagi.2021.635945

**Published:** 2021-04-27

**Authors:** Aparna Balagopalan, Benjamin Eyre, Jessica Robin, Frank Rudzicz, Jekaterina Novikova

**Affiliations:** ^1^Winterlight Labs Inc., Toronto, ON, Canada; ^2^Department of Computer Science, University of Toronto, Toronto, ON, Canada; ^3^Vector Institute for Artificial Intelligence, Toronto, ON, Canada; ^4^Unity Health Toronto, Toronto, ON, Canada

**Keywords:** Alzheimer's disease, dementia detection, MMSE regression, BERT, feature engineering, transfer learning

## Abstract

**Introduction:** Research related to the automatic detection of Alzheimer's disease (AD) is important, given the high prevalence of AD and the high cost of traditional diagnostic methods. Since AD significantly affects the content and acoustics of spontaneous speech, natural language processing, and machine learning provide promising techniques for reliably detecting AD. There has been a recent proliferation of classification models for AD, but these vary in the datasets used, model types and training and testing paradigms. In this study, we compare and contrast the performance of two common approaches for automatic AD detection from speech on the same, well-matched dataset, to determine the advantages of using domain knowledge vs. pre-trained transfer models.

**Methods:** Audio recordings and corresponding manually-transcribed speech transcripts of a picture description task administered to 156 demographically matched older adults, 78 with Alzheimer's Disease (AD) and 78 cognitively intact (healthy) were classified using machine learning and natural language processing as “AD” or “non-AD.” The audio was acoustically-enhanced, and post-processed to improve quality of the speech recording as well control for variation caused by recording conditions. Two approaches were used for classification of these speech samples: (1) using domain knowledge: extracting an extensive set of clinically relevant linguistic and acoustic features derived from speech and transcripts based on prior literature, and (2) using transfer-learning and leveraging large pre-trained machine learning models: using transcript-representations that are automatically derived from state-of-the-art pre-trained language models, by fine-tuning Bidirectional Encoder Representations from Transformer (BERT)-based sequence classification models.

**Results:** We compared the utility of speech transcript representations obtained from recent natural language processing models (i.e., BERT) to more clinically-interpretable language feature-based methods. Both the feature-based approaches and fine-tuned BERT models significantly outperformed the baseline linguistic model using a small set of linguistic features, demonstrating the importance of extensive linguistic information for detecting cognitive impairments relating to AD. We observed that fine-tuned BERT models numerically outperformed feature-based approaches on the AD detection task, but the difference was not statistically significant. Our main contribution is the observation that when tested on the same, demographically balanced dataset and tested on independent, unseen data, both domain knowledge and pretrained linguistic models have good predictive performance for detecting AD based on speech. It is notable that linguistic information alone is capable of achieving comparable, and even numerically better, performance than models including both acoustic and linguistic features here. We also try to shed light on the inner workings of the more black-box natural language processing model by performing an interpretability analysis, and find that attention weights reveal interesting patterns such as higher attribution to more important information content units in the picture description task, as well as pauses and filler words.

**Conclusion:** This approach supports the value of well-performing machine learning and linguistically-focussed processing techniques to detect AD from speech and highlights the need to compare model performance on carefully balanced datasets, using consistent same training parameters and independent test datasets in order to determine the best performing predictive model.

## 1. Introduction

Alzheimer's disease (AD) is a progressive neurodegenerative disease that causes problems with memory, thinking, and behavior. AD affects over 40 million people worldwide with high costs of acute and long-term care (Prince et al., 2016). Current forms of diagnosis are both time consuming and expensive (Prabhakaran et al., 2018), which might explain why almost half of those living with AD do not receive a timely diagnosis (Jammeh et al., 2018).

Studies have shown that valuable clinical information indicative of cognition can be obtained from spontaneous speech elicited using pictures (Goodglass et al., 2001). Studies have capitalized on this clinical observation, using speech analysis, natural language processing (NLP), and machine learning (ML) to distinguish between speech from healthy and cognitively impaired participants in datasets including semi-structured speech tasks such as picture description. Some of the first papers on this topic reported ML methods for automatic AD-detection using speech datasets achieving high classification performance (between 82 and 93% accuracy) (König et al., 2015; Fraser et al., 2016; Noorian et al., 2017; Karlekar et al., 2018; Zhu et al., 2018; Gosztolya et al., 2019). These models serve as quick, objective, and non-invasive assessments of an individual's cognitive status which could be developed into more accessible tools to facilitate clinical screening and diagnosis. Since these initial reports, there has been a proliferation of studies reporting classification models for AD based on speech, as described by recent reviews and meta-analyses (Slegers et al., 2018; de la Fuente Garcia et al., 2020; Petti et al., 2020; Pulido et al., 2020), but the field still lacks validation of predictive models on publicly-available, balanced, and standardized benchmark datasets.

The existing studies that have addressed differences between AD and non-AD speech and worked on developing speech-based AD biomarkers, are often descriptive rather than predictive. Thus, they often overlook common biases in evaluations of AD detection methods, such as repeated occurrences of speech from the same participant, variations in audio quality of speech samples, and imbalances of gender and age distribution in the used datasets, as noted in the systematic reviews and meta-analyses published on this topic (Slegers et al., 2018; Chen et al., 2020; Petti et al., 2020). As such, the existing ML models may be prone to the biases introduced in available data. In addition, the performance of the previously developed predictive AD-detection models has been evaluated using either random train/test split or a cross-validation technique, which may result in artificially increased reported performance of ML models (i.e., overfitting) as compared to their evaluation on a held out unseen dataset (more details on evaluation techniques are provided in the section 2.3.1.2), especially when it comes to smaller and unbalanced datasets (Johnson et al., 2018). Due to these reasons, it's difficult to compare model performance across papers and datasets, since they are rarely matched in terms of data and model characteristics.

To overcome the problem of bias and overfitting and introduce a common dataset to compare model performance, the ADReSS challenge (Luz et al., 2020) was introduced in 2020, in which the organizers provided an age/sex-matched balanced speech dataset, which consisted of speech from AD and non-AD participants describing a picture. The challenge consisted of two key tasks: (1) Speech classification task: classifying speech as AD or non-AD. (2) Neuropsychological score regression task: predicting Mini-Mental State Examination (MMSE) (Cockrell and Folstein, 2002) scores from speech. The organizers restricted access to the test dataset to make it completely unseen for participants to ensure the fair evaluation of models' performance. The work presented in this paper is focused entirely on this new balanced dataset and follows the ADReSS challenge's evaluation process. As such, the models presented in this paper are more generalizable to unseen data than those developed in the previously discussed studies.

In this work, we develop ML models to detect AD from speech using picture description data of the demographically-matched ADReSS Challenge speech dataset (Luz et al., 2020), and compare the following training regimes and input representations to detect AD:

1. **Using domain knowledge**: with this approach, we extract clinically relevant linguistic features from transcripts of speech, and acoustic features from corresponding audio files for binary AD vs. non-AD classification and MMSE score regression. The features extracted are informed by previous clinical and ML research in the space of cognitive impairment detection (Fraser et al., 2016).
2. **Using transfer learning**: with this approach, we fine-tune pre-trained BERT

We describe below the details of each approach.

### 1.1. Domain Knowledge-Based Approach

The overwhelming majority of NLP and ML approaches on AD detection from speech are still based on hand-crafted engineering of clinically-relevant features (de la Fuente Garcia et al., 2020). Previous work that focused on automatic AD detection from speech uses certain acoustic features (such as zero-crossing rate, Mel-frequency cepstral coefficients etc.) and linguistic features (such as proportions of various parts-of-speech (POS) tags (Orimaye et al., 2015; Fraser et al., 2016; Noorian et al., 2017), etc.) from speech transcripts. Fraser et al. (2016) extracted 370 linguistic and acoustic features from picture descriptions in the DementiaBank dataset, and obtained an AD detection accuracy of 82% at transcript-level. Fraser et al.'s model was evaluated using cross-validation. More recent studies showed the addition of normative data helped increase accuracy up to 93%, when evaluated using a random train/test split (Noorian et al., 2017; Balagopalan et al., 2018). Yancheva et al. (2015) showed ML models are capable of predicting the MMSE scores from features of speech elicited via picture descriptions, with mean absolute error of 2.91-3.83.

Detecting AD or predicting MMSE scores with pre-engineered features of speech and thereby infusing domain knowledge into the task has several advantages, such as more interpretable model decisions, the possibility to represent speech in different modalities (both acoustic and linguistic), and potentially lower computational resource requirements when paired with conventional ML models. However, there are also a few disadvantages, e.g., a feature engineering process is very expensive and time-consuming, it requires clinical expertise, is prone to biases in data, and carries the risk of missing highly relevant features.

### 1.2. Transfer Learning-Based Approach

In the recent years, transfer learning, or in other words, utilizing language representations from huge pre-trained neural models that learn robust representations for text, has become ubiquitous in NLP (Young et al., 2018). One of the most popular transfer learning models is BERT (Devlin et al., 2019), which trains “contextual embeddings” wherein a representation of a sentence (or transcript) is influenced by the context in which the words occur in sentences. This model offers enhanced parallelization and better modeling of long-range dependencies in text and as such, has achieved state-of-the-art performance on a variety of tasks in NLP. Previous research (Jawahar et al., 2019; Rogers et al., 2021) has suggested that it encodes language information (lexical, syntactic etc.) that is known to be important for performing complex natural language tasks, including AD detection from speech.

BERT uses powerful attention mechanisms to encode global dependencies between the input and output. This allows it to achieve state-of-the-art results on a suite of benchmarks (Devlin et al., 2019). Fine-tuning BERT for a few epochs can potentially attain good performance even on small datasets.

The transfer learning technique in general and BERT model specifically are promising approaches to apply to the task of AD detection from speech because such a technique eliminates the need of expensive and time-consuming feature engineering, mitigates the need of big training datasets, and potentially results in more generalizable models. However, the common critique is that BERT is pre-trained on the corpus of healthy language and as such is not usable for detecting AD. In addition, BERT is not directly interpretable, unlike feature-based models. Finally, the original version of the BERT model is only able to use text as input, thus eliminating the possibility to employ the acoustic modality of speech, when detecting AD. All these may be the reasons why BERT was not previously used for developing predictive models for AD detection, even though its performance on many other NLP tasks is exceptional.

### 1.3. Motivation and Contributions

Our motivation in this work is to benchmark a BERT training procedure on transcripts from a pathological speech dataset, and evaluate the effectiveness of high-level language representations from BERT in detecting AD. We are specifically interested in understanding whether BERT has a potential to outperform traditional widely used domain-knowledge based approaches given that it does not include acoustic features, and at the same time increase the generalizability of the predictive models.

To eliminate the biases of unbalanced data, we perform all our experiments on the carefully demographically-matched ADReSS dataset. To understand how well the presented models generalize to unseen data, we evaluate performance of the models using both cross-validation and testing on unseen held out dataset.

We find that the feature-based SVM model with RBF kernel outperforms all the other models, and performs on par with BERT, when evaluated using cross-validation. When evaluation is performed on the unseen held out test data, the fine-tuned BERT text sequence classification models achieve the highest AD detection accuracy of 83.3%. This BERT model numerically, though not significantly, outperforms the SVM model that achieves 81.3% accuracy on the unseen test set. These results show that: (1) Extensive feature-based—i.e., containing linguistic information for various aspects of language such as semantics, syntax, and lexicon—classification models significantly outperforms the linguistic baseline provided in the challenge showing that feature engineering to capture various aspects of language such as semantics and syntax helps with reliable detection of AD from speech, (2) BERT proved to be a generalizable model comparable to feature-based ones that make use of domain knowledge via hand-crafted feature engineering as shown by its higher performance on the independent test set in our case, (3) linguistic-only information encoded in BERT is sufficient for the strong predictive performance of the AD detection models.

## 2. Materials and Methods

### 2.1. ADReSS Dataset

Our data are derived from the ADReSS Challenge dataset (Luz et al., 2020), which consists of 156 speech recordings and associated transcripts from non-AD (*N* = 78) and AD (*N* = 78) English-speaking participants. Speech is elicited from participants through the Cookie Theft picture from the Boston Diagnostic Aphasia exam (Goodglass et al., 2001). Transcripts were annotated using the CHAT coding system (MacWhinney, 2000). In contrast to other speech datasets for AD detection such as DementiaBank's English Pitt Corpus (Becker et al., 1994), the ADReSS challenge dataset is carefully matched for age and gender in order to minimize risk of bias in the prediction tasks (Tables 1–3). Recordings were acoustically enhanced by the challenge organizers with stationary noise removal and audio volume normalization was applied across all speech segments to control for variation caused by recording conditions such as microphone placement (Luz et al., 2020). The speech dataset is divided into the train set and the unseen held out test set. MMSE (Cockrell and Folstein, 2002) scores are available for all but one of the participants in the train set.

**Table 1:** Basic characteristics of the patients in each group in the ADReSS challenge dataset are more balanced in comparison to DementiaBank.

**Dataset**			**Class**	
			**AD**	**Non-AD**
ADReSS	Train	Male	24	24
		Female	30	30
ADReSS	Test	Male	11	11
		Female	13	13
DementiaBank (Becker et al., 1994)	–	Male	125	83
		Female	197	146

**Table 2:** ADReSS Training set from Luz et al. (2020): basic characteristics of the patients in each group (M, male; F, female).

	**AD**			**Non-AD**		
**Age**	**M**	**F**	**MMSE (sd)**	**M**	**F**	**MMSE (sd)**
[50, 55)	1	0	30.0 (n/a)	1	0	29.0 (n/a)
[55, 60)	5	4	16.3 (4.9)	5	4	29.0 (1.3)
[60, 65)	3	6	18.3 (6.1)	3	6	29.3 (1.3)
[65, 70)	6	10	16.9 (5.8)	6	10	29.1 (0.9)
[70, 75)	6	8	15.8 (4.5)	6	8	29.1 (0.8)
[75, 80)	3	2	17.2 (5.4)	3	2	28.8 (0.4)
Total	24	30	17.0 (5.5)	24	30	29.1 (1.0)

**Table 3:** ADReSS test set from Luz et al. (2020): basic characteristics of the patients in each group (M, male; F, female).

	**AD**			**Non-AD**		
**Age**	**M**	**F**	**MMSE (sd)**	**M**	**F**	**MMSE (sd)**
[50, 55)	1	0	23.0 (n.a)	1	0	28.0 (n.a)
[55, 60)	2	2	18.7 (1.0)	2	2	28.5 (1.2)
[60, 65)	1	3	14.7 (3.7)	1	3	28.7 (0.9)
[65, 70)	3	4	23.2 (4.0)	3	4	29.4 (0.7)
[70, 75)	3	3	17.3 (6.9)	3	3	28.0 (2.4)
[75, 80)	1	1	21.5 (6.3)	1	1	30.0 (0.0)
Total	11	13	19.5 (5.3)	11	13	28.8 (1.5)

### 2.2. Feature Extraction

The speech transcripts in the dataset are manually transcribed as per the CHAT protocol (MacWhinney, 2000), and include speech segments from both the participant and an investigator. We only use the portion of the transcripts corresponding to the participant. Additionally, we combine all participant speech segments corresponding to a single picture description for extracting acoustic features.

We extract 509 manually-engineered features from transcripts and associated audio files (see Tables 4–6). These features are identified as indicators of cognitive impairment in previous literature, and hence encode domain knowledge.

**Table 4:** Summary of all lexico-syntactic features extracted.

**Feature type**	**#Features**	**Brief Description**
Syntactic complexity	36	L2 Analyzer features; utterance length, depth of syntactic parse tree
Production rules	104	Proportion of production type
Phrasal type ratios	13	Proportion, average length and rate of phrase types
Lexical norm-based	12	Average lexical norms across words for (e.g., imageability)
Lexical richness	6	Type-token ratios; brunet; Honor's statistic
Word category	5	Proportion of demonstratives, function words,
		Light verbs and inflected verbs, and propositions
Noun ratio	3	Ratios nouns:(nouns+verbs); nouns:verbs; pronouns:(nouns+pronouns)
Length measures	1	Average word length
Universal POS proportions	18	Proportions of Spacy universal POS tags
POS tag proportions	53	Proportions of Penn Treebank POS tags
Local coherence	15	Similarity between word2vec representations of utterances
Utterance distances	5	Fraction of pairs of utterances below a similarity threshold (0.5, 0.3, 0); avg/min distance
Speech-graph features	13	Representing words as nodes in a graph and computing density, number of loops, etc.
Utterance cohesion	1	Number of switches in verb tense across utterances divided by total number of utterances
Rate	2	Ratios—number of words: duration of audio; number of syllables: duration of speech,
Invalid words	1	Proportion of words not in the English dictionary
Sentiment norm-based	9	Average sentiment norms across all words, noun, and verbs

*The number of features in each subtype is shown in the second column (titled “#Features”)*.

**Table 5:** Summary of all acoustic/temporal features extracted.

**Feature type**	**#Features**	**Brief description**
Pauses and fillers	9	Total and mean duration of pauses; long and short pause counts;
		pause to word ratio; fillers (um, uh); duration of pauses to word durations
Fundamental frequency	4	Avg/min/max/median fundamental frequency of audio
Duration-related	2	Duration of audio and spoken segment of audio
Zero-crossing rate	4	Avg/variance/skewness/kurtosis of zero-crossing rate
MFCC	168	Avg/variance/skewness/kurtosis of 42 MFCC coefficients

*The number of features in each subtype is shown in the second column (titled “#Features”)*.

**Table 6:** Summary of all semantic features extracted.

**Feature type**	**#Features**	**Brief description**
Word frequency	10	Proportion of lemmatized words occurrences
Global coherence	15	Cosine distances between word2vec utterances and content units

*The number of features in each subtype is shown in the second column (titled “#Features”)*.

All the features are divided into three higher-level categories:

1. **Lexico-syntactic features (297):** Frequencies of various production rules from the constituency parsing tree of the transcripts (Chae and Nenkova, 2009), speech-graph based features (Mota et al., 2012), lexical norm-based features (e.g., average sentiment valence of all words in a transcript, average imageability of all words in a transcript; Warriner et al., 2013), features indicative of lexical richness. We also extract syntactic features (Ai and Lu, 2010) such as the proportion of various POS-tags, and similarity between consecutive utterances.
2. **Acoustic and temporal features (187):** Mel-frequency cepstral coefficients (MFCCs), fundamental frequency, statistics related to zero-crossing rate, as well as proportion of various pauses (for example, filled and unfilled pauses, ratio of a number of pauses to a number of words etc.; Davis and Maclagan, 2009).
3. **Semantic features based on picture description content (25):** Proportions of various information content units used in the picture, identified as being relevant to memory impairment in prior literature (Croisile et al., 1996).

### 2.3. Experiments

#### 2.3.1. AD vs. Non-AD Classification

##### 2.3.1.1. Training Regimes

We benchmark the following training regimes for classification: classifying features extracted at transcript-level and a BERT model fine-tuned on transcripts.

**Domain knowledge-based approach:** We classify lexicosyntactic, semantic, and acoustic features extracted at transcript-level with four conventional ML models (SVM), neural network (NN), random forest (RF), naïve Bayes (NB)^1^.

*Hyperparameter tuning:* All parameters in classification models were tuned to the best possible setting by searching within a grid of possible parameter values using 10-fold cross validation on the ADReSS challenge “train” set.

The random forest classifier fits 200 decision trees and considers $\sqrt{features}$ when looking for the best split. The minimum number of samples required to split an internal node is 2, and the minimum number of samples required to be at a leaf node is 2. Bootstrap samples are used when building trees. All other parameters are set to the default value.

The Gaussian Naive Bayes classifier is fit with balanced priors and variance smoothing coefficient set to 1*e* − 10 and all other parameters default in each case.

The SVM is trained with a radial basis function kernel with kernel coefficient(γ) 0.001, and regularization parameter set to 100.

The NN used consists of two layers of 10 units each (note we varied both the number of units and number of layers while tuning for the optimal hyperparameter setting). The ReLU activation function is used at each hidden layer. The model is trained using Adam (Kingma and Ba, 2014) for 200 epochs and with a batch size of number of samples in train set in each fold. All other parameters are default.

We perform feature selection by choosing top-k number of features, based on ANOVA *F*-value between label/features. The number of features is jointly optimized with the classification model parameters.

**Transfer learning-based approach:** In order to leverage the language information encoded by BERT (Devlin et al., 2019), we use pre-trained model weights to initialize our classification model. All our experiments are based on the *bert-base-uncased* variant (Devlin et al., 2019), which consists of 12 layers, each having a hidden size of 768 and 12 attention heads. Maximum input length is 512 tokens. Initial learning rate is set to 2*e* − 5, and Adam optimizer (Kingma and Ba, 2014) is used. Cross-entropy loss is used while fine-tuning for AD detection.

While the base BERT model is pre-trained with sentence pairs, our input to the model consists of speech transcripts with several transcribed utterances with start and separator special tokens from the BERT vocabulary at the beginning and end of each utterance respectively, following Liu and Lapata (2019). This is performed to ensure that utterance boundaries are easily encoded, since cross-utterance information such as coherence and utterance transitions is important for reliable AD detection (Fraser et al., 2016). An embedding, following Devlin et al. (2019), pooling information across all tokenized units in the transcript is extracted as the aggregate transcript representation from the BERT base for each transcript. This is then passed to the classification layer, and the combined model is fine-tuned on the AD detection task—all using an open-source PyTorch (Paszke et al., 2019) implementation of BERT-based text sequence classification models and tokenizers (Wolf et al., 2019). As noted by Devlin et al. (2019), this pooled embedding representation heavily depends on the fine-tuning task—in our case, AD detection at transcript level.

The transcript input to the classification model consists of several transcribed utterances with corresponding start and end tokens for each utterance, following (Liu and Lapata, 2019). The final hidden state corresponding to the first start (*[CLS]*) token in the transcript which summarizes the information across all tokens in the transcript using the self-attention mechanism in BERT is used as the aggregate representation, and passed to the classification layer (Devlin et al., 2019; Wolf et al., 2019). This model is then fine-tuned on training data.

*Hyperparameter tuning:* We optimize the number of epochs to 10 by varying it from 1 to 12 during CV. Adam optimizer (Kingma and Ba, 2014) and linear scheduling for the learning rate (Paszke et al., 2019) are used. Learning rate and other parameters are set based on prior work on fine-tuning BERT (Devlin et al., 2019; Wolf et al., 2019).

##### 2.3.1.2. Evaluation

**Cross-validation on ADReSS train set:** We use two CV strategies in our work—leave-one-subject-out CV (LOSO CV) and 10-fold CV at transcript level. We report evaluation metrics with LOSO CV for all models except fine-tuned BERT for direct comparison to challenge baselines. Due to computational constraints of GPU memory, we are unable to perform LOSO CV for the BERT model. Hence, we perform 10-fold CV to compare feature-based classification models with fine-tuned BERT. Values of performance metrics for each model are averaged across three runs with different random seeds in all cases.

**Predictions on ADReSS test set:** We generate three predictions with different seeds from each hyperparameter-optimized classifier trained on the complete train set, and then produce a majority prediction to avoid overfitting. We report performance on the challenge test set, as obtained from the challenge organizers. We evaluate task performance primarily using accuracy scores, since all train/test sets are known to be balanced. We also report precision, recall, specificity, and F1 with respect to the positive class (AD).

#### 2.3.2. MMSE Score Regression

##### 2.3.2.1. Training regimes

**Domain knowledge-based approach:** For this task, we benchmark two kinds of regression models, linear, and ridge, using pre-engineered features as input. MMSE scores are always within the range of 0–30, and so predictions are clipped to a range between 0 and 30.

*Hyperparameter tuning:* Each model's performance is optimized using hyperparameters selected via grid-search LOSO CV. We perform feature selection by choosing top-k number of features, based on an F-Score computed from the correlation of each feature with MMSE score. The number of features is optimized for all models. For ridge regression, the number of features is jointly optimized with the coefficient for L2 regularization, α.

##### 2.3.2.2. Evaluation

We report root mean squared error (RMSE) and mean absolute error (MAE) for the predictions produced by each of the models on the training set with LOSO CV. In addition, we include the RMSE for two models' predictions on the ADReSS test set. Hyperparameters for these models were selected based on performance in grid-search 10-fold cross validation on the training set, motivated by the thought that 10-fold CV better demonstrates how well a model will generalize to the test set.

## 3. Results

### 3.1. AD vs. Non-AD Classification

In Table 7, the classification performance with all the models evaluated on the train set via 10-fold CV is displayed. We observe that BERT numerically outperforms all domain knowledge-based ML models with respect to all metrics, with an average accuracy of 81.8%. SVM is the best-performing domain knowledge-based model. However, accuracy of the fine-tuned BERT model is not significantly higher than that of the SVM classifier based on an Kruskal-Wallis *H*-test (*H* = 0.4838, *p* > 0.05). Note that we used a Kruskal-Wallis *H*-test here, and in performance-comparisons in sections below since we observe that accuracy is not normally distributed on varying the random seed while training/inference.

**Table 7:** Ten-fold CV results averaged across three runs with different random seeds on the ADReSS train set.

**Model**	**#Features**	**Accuracy**	**Precision**	**Recall**	**Specificity**	**F1**
SVM	10	0.796	0.81	0.78	0.82	0.79
NN	10	0.762	0.77	0.75	0.77	0.76
RF	50	0.738	0.73	0.76	0.72	0.74
NB	80	0.750	0.76	0.74	0.76	0.75
BERT	–	**0.818**	**0.84**	**0.79**	**0.85**	**0.81**

*Accuracy for BERT is higher, but not significantly so from SVM (H = 0.4838, p > 0.05 Kruskal-Wallis H-test). Bold indicates the best result*.

We also report the performance of all our classification models with LOSO CV (**Table 9**). Each of our classification models significantly outperform the challenge baseline, which is uses 34 simple language summary statistic measures (e.g., duration, total utterances, MLU, type-token ratio, percentages of nine parts of speech) on the CHAT transcripts by a large margin (+10% accuracy for the best performing model, *p* = 0.036 with Kruskal-Wallis H = 4.35 test). Feature selection results in accuracy increase of about 13% for the SVM classifier.

Performance results on the unseen, held out challenge test set are shown in Table 8 and follow the trend of the cross-validated performance in terms of accuracy, with BERT outperforming the best feature-based classification model SVM with an accuracy of 83.33%, but not significantly so (*H* = 2.4, *p* > 0.05). The accuracy with a BERT-based classification model ranges between 85.14 and 81.25%.

**Table 8:** AD detection results on unseen, held out ADReSS test set averaged over three runs with different random seeds.

**Model**	**#Features**	**Accuracy**	**Precision**	**Recall**	**Specificity**	**F1**	**AUROC**
Baseline (Luz et al., 2020)	–	0.7500	–	–	–	0.7800	–
SVM	10	0.8125	0.8000	0.8333	0.7917	0.8124	0.8125
NN	10	0.7708	0.7671	0.7778	0.7639	0.7708	0.7708
RF	50	0.7569	0.8033	0.6806	0.8333	0.7555	0.7500
NB	80	0.7292	0.7895	0.6250	0.8333	0.7262	0.7292
BERT	–	**0.8332**	**0.8389**	**0.8333**	**0.8333**	**0.8327**	**0.8333**

*Bold indicates the best result*.

### 3.2. MMSE Score Regression

Performance of regression models evaluated on both train and test sets is shown in Table 9. Ridge regression with 25 features selected attains the lowest RMSE of 4.56 (with a corresponding MAE of 3.50, or 11.67% error) during LOSO-CV on the training set. The results show that feature selection is impactful for performance and helps achieve a decrease of up to 1.5 RMSE points (and up to 0.86 of MAE) for a ridge regressor. Furthermore, a ridge regressor is able to achieve an RMSE of 4.56 on the ADReSS test set, a decrease of 0.64 from the baseline. We also experimented with different non-linear regression methods—however, given the small dataset size and the difficulty of the task, the linear regression models highlighted in Table 9 performed the best.

**Table 9:** LOSO-CV MMSE regression results on the ADReSS train and test sets.

**Model**	**#Features**	**α**	**RMSE**	**MAE**	**RMSE**
			**Train set**		**Test set**
Baseline (Luz et al., 2020)	–	–	4.38		5.20
LR	15	–	5.37	4.18	4.94
LR	20	–	4.94	3.72	–
Ridge	509	12	6.06	4.36	–
Ridge	35	12	4.87	3.79	**4.56**
Ridge	25	10	**4.56**	**3.50**	–

*Bold indicates the best result*.

## 4. Discussion

### 4.1. Feature Differentiation Analysis

While we extracted a large number of linguistic and acoustic features to capture a wide range of linguistic and acoustic changes in speech associated with AD, based on a survey of prior literature (Yancheva et al., 2015; Fraser et al., 2016; Pou-Prom and Rudzicz, 2018; Zhu et al., 2019), we are also interested in identifying the *most differentiating* features between AD and non-AD speech. In order to study statistically significant differences in linguistic/acoustic phenomena, we perform independent *t*-tests between feature means for each class in the ADReSS training set, following the methodology followed by Eyre et al. (2020). 87 features are significantly different between the two groups at *p* < 0.05. Seventy-nine of these are text-based lexicosyntactic and semantic features, while eight are acoustic. These eight acoustic features include the number of long pauses, pause duration, and mean/skewness/variance-statistics of various MFCC coefficients. However, after Bonferroni correction for multiple testing, we identify that only 13 features are significantly different between AD and non-AD speech at *p* < 9*e* − 5, and none of these features are acoustic (Table 10). This implies that linguistic features are particularly differentiating between the AD/non-AD classes here, which explains why models trained only on linguistic features (i.e., BERT models) attain performance well above random chance.

**Table 10:** Feature differentiation analysis results for the most important features, based on ADReSS train set.

**Feature**	**Feature type**	**μ_*AD*_**	**μ_*non*−*AD*_**	**Correlation**	**Weight**
Average cosine distance between utterances	Semantic	0.91	0.94	–	–
Fraction of pairs of utterances below a similarity threshold (0.5)	Semantic	0.03	0.01	–	–
Cosine distance between word2vec utterances and content units	Semantic	0.46	0.38	−0.54^*^	−1.01
Distinct content units mentioned: total content units	Semantic	0.27	0.45	0.63^*^	1.78
Distinct action content units mentioned: total content units	Semantic	0.15	0.30	0.49^*^	1.04
Distinct object content units mentioned: total content units	Semantic	0.28	0.47	0.59^*^	1.72
Cosine distance between GloVe utterances and content units	Semantic	–	–	−0.42^*^	−0.03
Average word length (in letters)	Lexico-syntactic	3.57	3.78	0.45^*^	1.07
Proportion of pronouns	Lexico-syntactic	0.09	0.06	–	–
Ratio (pronouns):(pronouns+nouns)	Lexico-syntactic	0.35	0.23	–	–
Proportion of personal pronouns	Lexico-syntactic	0.09	0.06	–	–
Proportion of adverbs	Lexico-syntactic	0.06	0.04	−0.41^*^	−0.41
Proportion of adverbial phrases amongst all rules	Lexico-syntactic	0.02	0.01	−0.37	−0.74
Proportion of non-dictionary words	Lexico-syntactic	0.11	0.08	–	–
Proportion of gerund verbs	Lexico-syntactic	–	–	0.37	1.08
Proportion of words in adverb category	Lexico-syntactic	–	–	−0.4^*^	−0.49

*μ_AD_ and μ_non−AD_ show the means of the 13 significantly different features at p < 9e-5 (after Bonferroni correction) for the AD and non-AD group, respectively. We also show Spearman correlation between MMSE score and features, and regression weights of the features associated with the five greatest and five lowest regression weights from our regression experiments*.

* *Next to correlation indicates significance at p < 9e-5*.

The features that differentiate the AD and non-AD groups largely indicate semantic impairments in AD, reflected in the types of words used and the content of their picture descriptions. Importantly, many of the differentiating features replicate findings from Fraser et al. (2016), suggesting that despite the present dataset being more demographically balanced, many of the previous findings maintain. In addition, the differentiating features are consistent with other previous clinical literature documenting decreased specificity and information content in AD. For example, the features relating to the content units in the picture and the cosine similarity between utterances and picture content units show that the picture descriptions produced in AD have fewer relevant content words and that the words used are less semantically related to the themes of the picture. Lower average cosine distance in AD signifies more repetition in speech. These findings are consistent with previous studies reporting reduced information content and coherence in AD (Croisile et al., 1996; Snowdon et al., 1996; Dijkstra et al., 2004; Forbes-McKay and Venneri, 2005; Riley et al., 2005; Le et al., 2011; Ahmed et al., 2013; Boschi et al., 2017). Other differentiating features related to the use of shorter words, and increased use of pronouns, adverbs, and words not found in the dictionary. These features may all reflect the use of less specific and simpler language, and replicate previous findings of decreased specificity of language in AD (Le et al., 2011; Ahmed et al., 2013; Szatloczki et al., 2015; Fraser et al., 2016). Interestingly, while Fraser et al. (2016) found differences in acoustic features, none of those findings survived Bonferroni correction in the present study, which may indicate that this age/sex-balanced dataset reduced the acoustic differences between groups.

**Figure 1:**
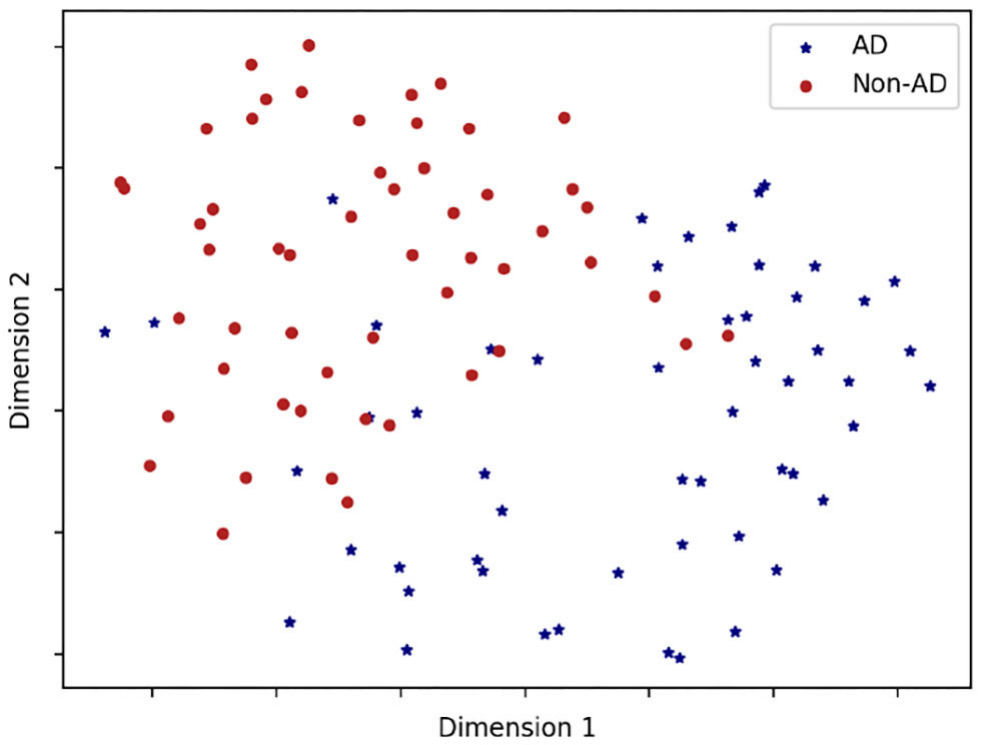
A t-SNE plot showing class separation. Note we only use the 13 features significantly different between classes (see Table 10) in feature representation for this plot.

In order to visualize the class-separability of the feature-based representations, we visualize (t-SNE) t-Distributed Stochastic Neighbor Embedding (Maaten and Hinton, 2008) plots in Figure 1. t-SNE is a non-linear dimensionality reduction algorithm used for exploring high-dimensional data. It maps multi-dimensional data to two or more dimensions suitable for human observation. We observe strong class-separation between the two classes, indicating that a non-linear model would be capable of good AD detection performance with these representations.

### 4.2. Interpreting Attention Patterns in BERT-Based Models

**Figure 2:**
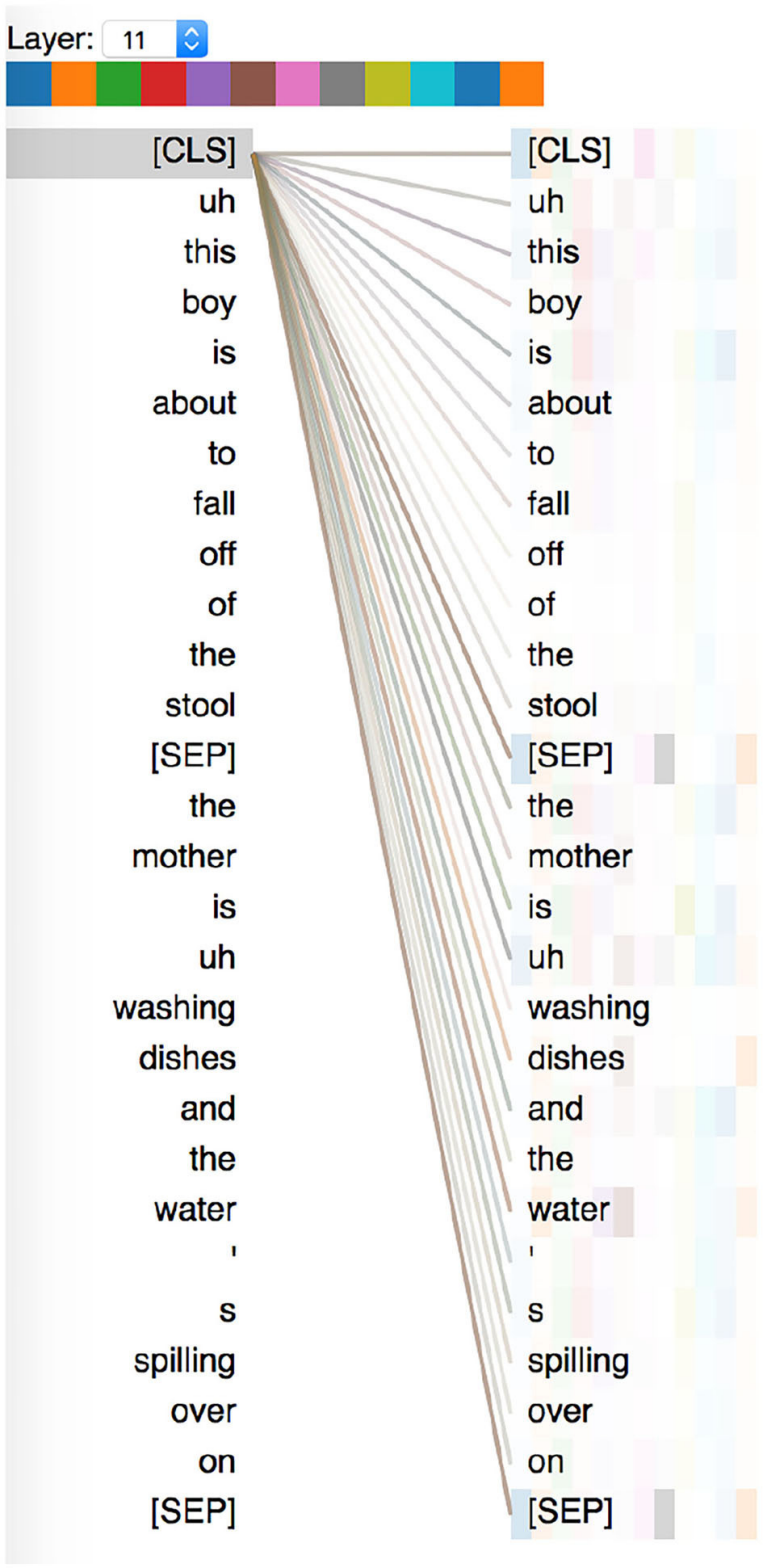
An attention visualization plot showing attention contributions of embeddings corresponding to each word to the “pooled” representation. This example is a sub-sample (first two utterances) of a speech transcript from a healthy person.

We look at multi-scale attention visualizations of BERT fine-tuned for the AD detection task, using the BertViz library (Vig, 2019) (Figure 2). Self-attention is an important component of BERT-based models, and looking at attention patterns can help us interpret model decisions. We used the BERT-base model which consists of 12 layers, and 12 attention heads in each layer. We visualize, for both AD and healthy speech transcripts, the attention weights for the final “[CLS]” token, whose representation is passed to the fully-connected layer for classification. On analyzing the attention weights attributed to words in both healthy and AD transcripts, we find that:

1. attention weights are often attributed to a few important “information content units.” which have been identified to be important speech indicators of AD in prior work (Fraser et al., 2016) such as “water,” “boy,” etc.
2. attention weights are also sometimes attributed to pauses and fillers, such as “uh” and “um.”
3. attention weights are also attributed to the sentence separator tokens, and we think this approximates to roughly counting the number of utterances in the transcript.

Hence, as seen in sections 4.1 and 4.2, we observe that for both the feature-based classification models and BERT-based models, information units and fillers such as “uh” and “um” seem to be important predictors, similar to findings observed by Yuan et al. (2020).

### 4.3. Analysing AD Detection Performance Differences

We observe that both feature-based and BERT-based classification models are significantly better than the linguistic baseline, showing the importance of an extensive amount of linguistic features for detecting AD-related differences. When compared on this well-matched dataset, BERT tended to have higher performance, but the difference was not significant. Based on feature differentiation analysis, we hypothesize that good performance with a text-focused BERT model on this speech classification task is due to the strong utility of linguistic features on this dataset. BERT captures a wide range of linguistic phenomena due to its training methodology, potentially encapsulating most of the important lexico-syntactic and semantic features. It is thus able to use information present in the lexicon, syntax, and semantics of the transcribed speech after fine-tuning (Jawahar et al., 2019).

We also see a trend of better performance when increasing the number of folds (see SVM in Tables 7, 11) in cross-validation. We postulate that this is due to the small size of the dataset, and hence differences in training set size in each fold (*N*_*train*_ = 107 with LOSO, *N*_*train*_ = 98 with 10-fold CV). Note that, in this dataset, both feature-based and BERT-based classification methods rely on linguistic features to achieve better classification than baseline. This implies that the linguistic features from speech transcripts are quite informative for the AD detection task. Hence, an interesting direction of future research is expanding our current set of features to incorporate more discourse-related features (which could be getting captured to some degree in fine-tuned BERT models).

**Table 11:** LOSO-CV results averaged across three runs with different random seeds on the ADReSS train set.

**Model**	**#Features**	**Accuracy**	**Precision**	**Recall**	**Specificity**	**F1**
Baseline (Luz et al., 2020)	–	0.768	0.77	0.76	–	0.77
SVM	509	0.741	0.75	0.72	0.76	0.74
SVM	10	**0.870**	**0.90**	**0.83**	**0.91**	**0.87**
NN	10	0.836	0.86	0.81	0.86	0.83
RF	50	0.778	0.79	0.77	0.79	0.78
NB	80	0.787	0.80	0.76	0.82	0.78

*Accuracy for SVM is significantly higher than NN (H = 4.50, p = 0.034 Kruskal-Wallis H-test). Bold indicates the best result*.

### 4.4. Regression Weights for MMSE Prediction

To assess the relative importance of individual input features for MMSE prediction, we report features with the five highest and five lowest regression weights reflecting the five strongest positive and negative relationships with MMSE scores (Table 10). Each presented value is the average weight assigned to that feature across each of the LOSO CV folds. We also present the correlation with MMSE score coefficients for those 10 features, as well as their significance, in Table 10. We observe that for each of these highly weighted features, a positive or negative correlation coefficient is accompanied by a positive or negative regression weight, respectively. This demonstrates that these 10 features are so distinguishing that, even in the presence of other regressors, their relationship with MMSE score remains the same. We also note that all 10 of these are linguistic features, further demonstrating that linguistic information is particularly distinguishing when it comes to predicting the severity of a patient's AD. Notably, seven of the ten features were among those that differentiated between AD and non-AD groups, demonstrating that there is high overlap between the features relevant to group differentiation and MMSE score prediction. These features included those relating to the information content and the coherence of picture descriptions, reflected by content unit and cosine distance features. Word length and use of adverbs were also relevant to MMSE prediction, with longer words and fewer adverbs correlating with higher MMSE scores. The use of gerund verbs was found to have a high regression weight for MMSE prediction and positively correlated with MMSE scores, despite not being significantly different between AD and non-AD groups after Bonferroni correction. Reduced use of inflected verbs has been found in some previous research (Ahmed et al., 2013; Fraser et al., 2016), and is thought to reflect an grammatic impairment.

## 5. Conclusions

In this paper, we rigorously compare two widely used approaches—linguistic and acoustic feature engineering based on domain knowledge, and text-only transfer learning using fine-tuned BERT classification model. Our results show that pre-trained models that are fine-tuned for the AD classification task are capable of performing well on AD detection, achieving comparable, or even slightly improved performance compared to hand-crafted feature engineering. We observe that linguistic features are capable of attaining predictive performance well above chance on this acoustically and demographically balanced speech dataset, and posit this to be the reason why a text-only approach with BERT numerically outperforms a multi-modal feature-engineering based approach. The present findings highlight the importance of measuring the linguistic, and especially semantic content of speech, in addition to acoustic analyses. In future work, it would be interesting to study methods that combine feature-based and pre-trained neural LM-based prediction models to optimize AD detection from speech—this could potentially help harness complementary benefits of both approaches. It is interesting to note that the winners of the ADReSS challenge also used a pre-trained language model, augmented with additional information about speech disfluencies (Yuan et al., 2020), which outperforms our best model by 6% in accuracy and F1-score, further indicating the degree of promise in such an approach. These results build on previous work to demonstrate how automated speech analysis can be used to help characterize AD. Speech samples can be collected quickly and non-invasively, and as demonstrated in the present results, yield measures relating to the presence and severity of AD.

Further work will build on these results to develop improved tools for disease screening and monitoring in AD, improving the efficiency of clinical research and treatment. In the future, we will experiment with different neural models such as XLNet (Yang et al., 2019), and with different tokenization and encoding strategies for transcript representations. A direction for future work is developing ML models that combine representations from BERT and hand-crafted features (Yu et al., 2015). Such feature-fusion approaches could potentially boost performance on the cognitive impairment detection task.

## Data Availability Statement

Publicly available datasets were analyzed in this study. This data can be found at: https://dementia.talkbank.org/.

## Ethics Statement

The studies involving human participants were reviewed and approved by DementiaBank consortium. The patients/participants provided their written informed consent to participate in this study.

## Author Contributions

All authors contributed to writing and edits. Methods and analyses were performed by AB, JN, and BE.

## Conflict of Interest

Authors AB, BE, JR and JN were employed by company Winterlight Labs Inc. The remaining author declares that the research was conducted in the absence of any commercial or financial relationships that could be construed as a potential conflict of interest.

## Footnotes

^1^https://scikit-learn.org/stable/.
